# Metataxonomic Analysis of Bacteria Entrapped in a Stalactite’s Core and Their Possible Environmental Origins

**DOI:** 10.3390/microorganisms9122411

**Published:** 2021-11-23

**Authors:** George Michail, Lefkothea Karapetsi, Panagiotis Madesis, Angeliki Reizopoulou, Ioannis Vagelas

**Affiliations:** 1 Department of Ichthyology and Aquatic Environment, School of Agricultural Sciences, University of Thessaly, 38446 Volos, Greece; 2 Laboratory of Molecular Biology of Plants, Department of Agriculture Crop Production and Rural Environment, School of Agricultural Sciences, University of Thessaly, 38446 Volos, Greece; lefki8@certh.gr (L.K.); pmadesis@uth.gr (P.M.); 3 Centre for Research and Technology (CERTH), Institute of Applied Biosciences (INAB), 57001 Thessaloniki, Greece; 4 Volos Natural History Museum, 38333 Volos, Greece; reizopoulou@gmail.com; 5 Department of Agriculture Crop Production and Rural Environment, University of Thessaly, 38446 Volos, Greece; vagelas@uth.gr

**Keywords:** environmental microbiology, cave microbiology, bacterial community, rare microbial taxa-biosphere, 16S rRNA

## Abstract

Much is known about microbes originally identified in caves, but little is known about the entrapment of microbes (bacteria) in stalactites and their possible environmental origins. This study presents data regarding the significant environmental distribution of prokaryotic bacterial taxa of a Greek stalactite core. We investigated the involvement of those bacteria communities in stalactites using a metataxonomic analysis approach of partial 16S rRNA genes. The metataxonomic analysis of stalactite core material revealed an exceptionally broad ecological spectrum of bacteria classified as members of Proteobacteria, Actinobacteria, Firmicutes, Verrucomicrobia, and other unclassified bacteria. We concluded that (i) the bacterial transport process is possible through water movement from the upper ground cave environment, forming cave speleothems such as stalactites, (ii) bacterial genera such as *Polaromonas*, *Thioprofundum,* and phylum *Verrucomicrobia* trapped inside the stalactite support the paleoecology, paleomicrobiology, and paleoclimate variations, (iii) the entrapment of certain bacteria taxa associated with water, soil, animals, and plants such as Micrococcales, Propionibacteriales, Acidimicrobiales, Pseudonocardiales, and α-, β-, and γ-Proteobacteria.

## 1. Introduction

Caves represent a distinctive ecosystem on Earth and compose unique underground microbial communities such as bacteria. Cave and bio-speleological research has been increased in the last thirty years, and available literature indicates that the presence of bacterial and fungal colonization is relatively extensive. Caves generally lack rich fungal diversity, while cave bacterial diversity and biomass are much higher [1]. It appears that most fungal and bacterial taxa reported in caves are generally discovered within the surrounding environment above ground [1]. Mason-Williams and Benson-Evans [2] reported that the bacteria within the air of numerous smaller British caves were directly associated with the frequency of outside cave air disturbances.

It has been demonstrated that the variation and composition of the ancient microbial communities may be potentially reflective of paleo-environmental changes [3,4,5]. Seasonal variation within the composition and the diversity of bacteria in trickling waters might indicate a high possibility of exploiting ancient microbial DNA in karst ecosystems to reconstruct paleo-environmental changes [6].

Speleothems (e.g., stalagmites, stalactites, and flowstones) discovered in caves around the globe are a natural library of paleorainfall as they are molded by groundwater, seawater, and downfall, respectively. They contain tiny quantities of water which are entrapped in so-called fluid inclusions, analogous to gas bubbles in ice cores, within and between the calcite crystals. Downpour water above the cave area infiltrates into the soil and epikarst and will convey through micro-cracks to eventually drop from soda straws hanging from the roof of the cavern onto a stalagmite. It is challenging to find direct and unaltered water, disseminated from past precipitation in mainland areas and in caverns worldwide, capable of producing stalactites and stalagmites that embody fossil water and entrap bacteria that is well-preserved for millennia in a very natural environment [7]. In nature, fluid additions in halite, gypsum, and other salts can maintain both living cells and DNA, therefore inheriting genetic records of the microbial pluralism and environmental circumstances of over nearly 150 million years [8].

Thus far, very little work has been performed on the microbes found in stalactites. Such published articles demonstrated that microbes might play a critical role in speleothem growth [9,10,11,12,13].

Studies on cave microbiology generally investigate the microbiological heterogeneity in the cave and the metabolic capabilities of these microbes [14]. Moreover, because caverns are defined by an absence of light, low to moderate temperatures, high moisture, and scarce nutrients, they can be distinguished from the land surface and deeper substrates [15]. Μicrobes growing in these ecosystems such as bacteria are limited in general to Proteobacteria, which are most predominant in water and air samples, and to Actinobacteria, which are predominant in sediment and rock samples [16]. Recent data showed that the cave sediments, as oligotrophic environments, harbor high phylogenetic diversity dominated by Actinobacteria and Proteobacteria. De Mandal et al. [17] suggested that bacteria represent a considerable part of a cave’s biodiversity, playing a key role in preserving a cave’s ecosystem. Many researchers mention that underground stalactites may play the role of natural traps for microorganisms within the fissure water [12,18].

The use of genetics in paleomicrobiology nearly fully obscures historical methods, such as microscopy, to detect or characterize ancient microbes. The capability to examine an amalgam of genetic material from all of the microorganisms present in a sample (the microbiome) using metataxonomic techniques has also resulted in revived interest and profound analytical and technical breakthroughs in the paleomicrobiology field [19,20,21,22]. Based on the above, we provide evidence that the bacteria detected in the stalactite core existed at some point in time above or around (upper ground environment) the cave’s ecosystem and were transferred and encapsulated in the stalactite core possibly through water flow.

## 2. Material and Methods

### 2.1. Study Site

The Agios Athanasios cave is located in East-Central Greece (39°28′43.9″N, 22°52′53.9″ E, 400 m above sea level) and developed as a 200 m long horizontal maze of passages. The depth of the cave is 20–30 m below the surface, and the light intensity level inside the cavern was zero. The region around the cave is covered by Carla’s Lake water, and *Quercus coccifera* is a dominant species of the peninsula land.

### 2.2. Sample Collection

**Figure 1:**
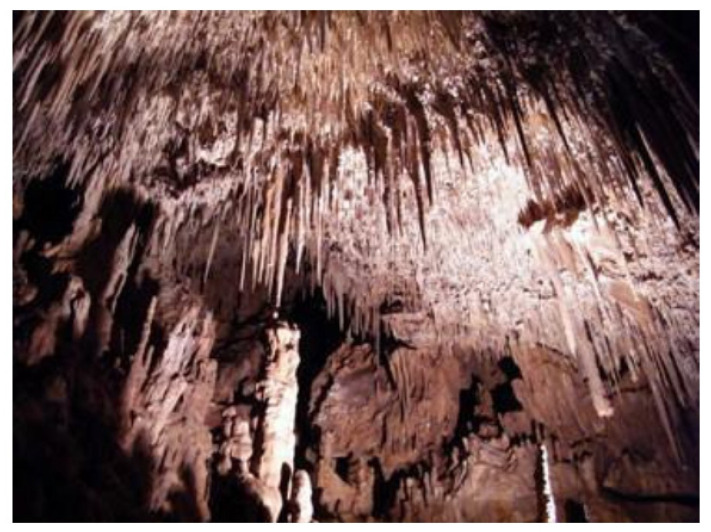
Formation of Stalactites inside cave Agios Athanasios, Central Thessaly, Greece.

For this study, we used subsamples from a stalactite collected using gloves in a plastic sterile container that is symmetrically shaped and has a diameter of 7.5–8 cm. The stalactite sample was collected in 2021 and was more than 50 m deep inside the Agios Athanasios cave (Figure 1). In this paper, we present taxonomic results obtained by metataxonomics (16S rRNA gene sequencing) from only one stalactite sample and not more, as there are no statistically significant changes obtained among phyla, families, and genera, as presented by Durazzi et al. [23].

### 2.3. DNA Extraction and DNA Sequence Analysis

The total metataxonomic DNA from a stalactite sample was extracted immediately after collection using a NucleoSpin Soil DNA isolation kit (Macherey-Nagel, Duren, Germany) according to the manufacturer’s instructions. Four replications (S1, S2, S3, S4; Supplementary Materials Figures S1–S10) of the same core sample were used for the DNA extraction with different parameters. The DNA quality and concentration were measured by Quawell UV-Vis Spectrophotometer (Q5000). The DNA samples were stored at −20 °C until the library preparation was performed.

Stalactite core samples were sequenced at the Institute of Applied Biosciences. Illumina’s 16S Metataxonomics Protocol (Part # 15044223 Rev. B) was applied for the amplification of V3 and V4 regions of the bacterial 16S rRNA gene using the following primers containing the Illumina overhang adapters. The forward and reverse universal primer sequences, as reported by Klindworth et al. [24], are, respectively, 5′-TCGTCGGCA GCGTCAGATGTGTATAAGAGACAGCCTACGGGNGGCWGCAG-3′ and 5′-GTCTCGTG GGCTCGGAGATGTGTATAAGAGACAGGACTACHV GGG-TATCTAATCC-3′ and create an amplicon of approximately 460 bp. For PCR amplification, 2× Kapa HiFi Hot-Start ReadyMix was used, and the PCR products were then cleaned using Beckman Coulter Agencourt AMPure XP Beads according to the 16S Meta-genomics protocol. The indices and Illumina sequencing adapters were attached using the Nextera^®^ XT Index Kit, followed by a second clean-up with Beckman Coulter Agencourt AMPure XP Beads. The libraries’ quantification was performed using a Qubit™ 3.0 Fluorometer (Life Technology Ltd., Paisley, UK). Moreover, 2 μL of the final libraries was run on a Fragment Analyzer with the method DNF-473-33-SS NGS Fragment 1–6000 bp in order to check the quality and verify the size of the libraries. After library quantification, normalization, and pooling, the concentrations were adjusted to 125 pM and prepared for loading on the Illumina MiSeq according to Illumina’s 16S Metataxonomics Protocol (Part # 15044223 Rev. B). The libraries’ pool was denatured and loaded on the Illumina MiSeq at 12.5 pM and sequenced paired-end (2 × 300) using a MiSeq^®^ Reagent Kitv3 (600 cycles) (Illumina, Inc., San Diego, CA, USA).

Furthermore, another important issue that needs to be addressed is that inside the stalactite core, the abundance of the bacteria entrapped is scarce with a lower number of reads (less than 500,000) compared to other biological samples (i.e., gastrointestinal tract with up to 2^19^ reads). It is obvious that in this case, the use of metataxonomics (16S DNA) is favorable and performs similarly or even better than other metagenomics (i.e., whole shotgun metagenomic sequencing) (Durazzi et al., 2021) [23].

### 2.4. Bioinformatics and Statistical Analysis

The raw sequence data were processed using the Mothur pipeline [25], performing the Standard Operating Procedure (SOP) for MiSeq data, developed by the creators of the Mothur software package, the Schloss lab, within Galaxy [26,27]. First, the 300 bp paired-end reads were organized into a paired collection and forward and reverse reads for each sample were combined into a single FASTA file. Second, the quality control and the data cleaning were performed to remove sequences with ambiguous bases and anything longer than 460 bp. The optimization of the files for computation was performed to remove duplicate sequences. Third, the sequence reads were aligned against the reference alignments of the Silva database (sil-va.seed_v138_1.align) using the Needleman alignment method and the k-mer search method. Further de-noising of the sequences was performed by pre-clustering and sorting the sequences by abundance. Finally, sequencing artifacts known as chimeras were removed, as well as non-bacterial sequences, including Archaea, Eukaryota, and unknown. Operational taxonomic units (OTUs) were identified at the 97% DNA similarity level and at the Order tax level. We considered 5516 OTUs associated with bacteria. For Alpha diversity, rarefaction curves describing the number of OTUs observed as a function of sampling effort were generated. Beta diversity metrics (Bray–Curtis) were used to measure the bacterial community composition. A 4-way Venn diagram and a table listing the shared OTUs were also generated. External tools were used to visualize the results of our analyses: the Krona tool for pie charts, phyloseq (R package) and PowerBI for abundance.

In order to determine the statistical significance of clustering, a principal coordinate analysis (PCoA) and an NMDS showed the similarity in OTU abundance profiles between samples and the quality of the ordination, respectively. To test which OTUs are responsible for shifting the samples along the two axes, we measured the correlation of the relative abundance of each OTU with the two axes in the NMDS dataset.

All above data sets are available as Supplementary Material as: OTU; Class (16S-S1, 16S-S2, 16S-S3, 16S-S4); Family (16S-S1, 16S-S2, 16S-S3, 16S-S4); Genus (16S-S1, 16S-S2, 16S-S3, 16S-S4); Sample (Abundance—Phylum); Sample (Abundance—Class); Alpha diversity; Bray–Curtis Heatmap; Venn diagram; Krona pie chart, (Supplementary Figures S1–S10).

## 3. Results

### 3.1. Bacterial Communities Encapsulated during the Formation of Stalactite

**Figure 2:**
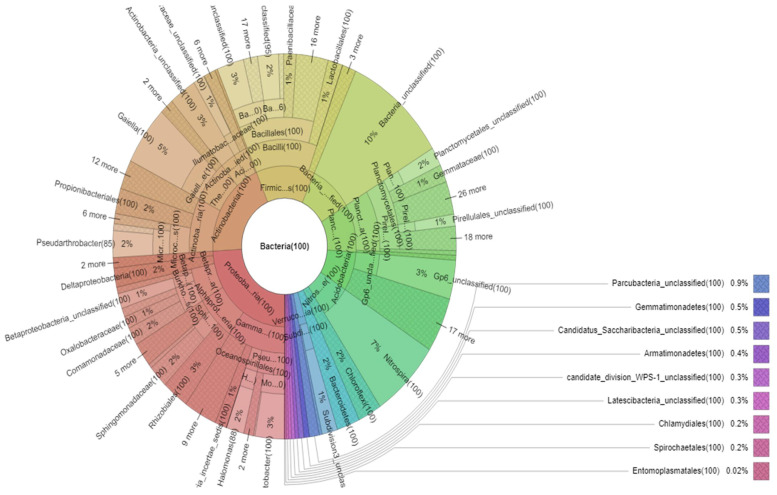
Average of bacteria composition of cave stalactite core sample at phyla and family levels from a total of 352,637 reads.

**Figure 3:**
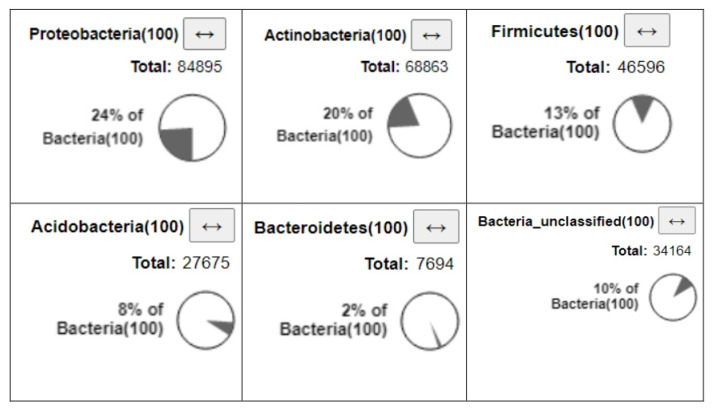
Total number of reads and percent of bacteria diversity of stalactites homogenized cores samples at phyla level.

Figure 2 presents the bacterial communities recorded from a cave stalactite core in central Greece. The percent of bacteria communities’ compositions at the phylum level from a cave stalactite core are presented in Figure 3. The bacteria metataxonomics analysis yielded a total of 352,637 reads. Within the bacteria, most phylotypes were classified under the phyla Proteobacteria (24% of bacteria) with an average of 84,895 reads, Actinobacteria (20% of bacteria) with an average of 68,863 reads, Firmicutes (13% of bacteria) with an average of 46,596 reads, Acidobacteria (8% of bacteria) with an average of 27,675 reads, Bacteroidetes (2% of bacteria) with an average of 7694 reads, and Bacteria unclassified (10% of bacteria) with an average of 34,164 reads, as shown in Figure 3.

### 3.2. Proteobacteria

Within the Proteobacteria, most phylotypes were classified under the classes *α*-Proteobacteria (28% of Proteobacteria) with an average of 23,934 reads, *β*-Proteobacteria (21% of Proteobacteria) with an average of 17,931 reads, and *γ*-Proteobacteria (40% of Proteobacteria) with an average of 34,298 reads (Table 1). Under the phylum Proteobacteria, 18 families were recorded. In detail, four families (*Sphingomonadaceae*, *Bradyrhizobiaceae*, *Hyphomicrobiaceae*, and *Rhizobiaceae*) were recorded under the class *α*-Proteobacteria; three families (*Oxalobacteraceae*, *Comamonadaceae*, *Burkholderiaceae*) were recorded under the class *β*-Proteobacteria, and eleven families (*Moraxellaceae*, *Pseudomonadaceae*, *Halomonadaceae*, *Xanthomonadaceae*, *Rhodanobacteraceae*, *Enterobacteriaceae*, *Yersiniaceae*, *Erwiniaceae*, *Steroidobacteraceae*, *Nevskiaceae* and *Thioprofundaceae*) were recorded under the class *γ*-Proteobacteria (Table 1). The dominant genus under the class *α*-*β* and *γ*-Proteobacteria (Table 1) were: (*Sphingomonas* and *Pedomicrobium*), (*Massilia*, *Polaromonas* and *Ralstonia*), and (*Acinetobacter*, *Pseudomonas*, *Halomonas*, *Lysobacter*, *Escherichia*/*Shigella*, *Panacagrimonas* and *Thioprofundum*), respectively. However, no genus was identified under the *families Bradyrhizobiaceae*, *Rhizobiaceae*, *Rhodanobacteraceae*, and *Steroidobacteraceae* (Table 1). The most dominant genus was *Sphingomonas* (5260 reads), a genus that is widely distributed in nature and has been isolated from many different aquatic and terrestrial habitats, as well as plant root systems, clinical specimens, and other sources (Table 1).

### 3.3. α-Proteobacteria

Under the class *α*-Proteobacteria, most phylotypes were classified under the orders Rhizobiales, Sphingomonadales, and Rhodospirillales with averages of 8902, 7940, and 2279 reads, respectively. Under the order Rhizobiales, the most dominant families were Bradyrhizobiaceae, Hyphomicrobiaceae, and Rhizobiaceae with averages of 2784, 1675, and 1038 reads, respectively. The presence of nitrogen-fixing Rhizobiales, common inhabitants of the legume roots, suggests that these bacteria were transferred and deposited in the stalactite a long time ago, signifying that the upper ground cave area was fully covered by legume plants and that the migration from roots to stalactites was achieved.

### 3.4. β-Proteobacteria

Under the class *β*-Proteobacteria, most phylotypes were classified under the order Burkholderiales and the order Nitrosomonadales, with averages of 11,533 and 1937 reads, respectively. Under the order Burkholderiales, the most dominant families were Comamonadaceae, Oxalobacteraceae, and Burkholderiaceae with averages of 5368, 3551, and 1462 reads, respectively. Under the order Burkholderiales and the family Burkholderiaceae, the genus *Ralstonia* was classified with an average of 120 reads. Under the order Nitrosomonadales (1937 reads) and the family Nitrosomonadaceae (1359 reads), the most dominant genera were *Nitrospira* (1066 reads) and *Nitrosomonas* with an average of 293 reads.

### 3.5. γ-Proteobacteria

Under the class *γ*-Proteobacteria, most phylotypes were classified under the orders Pseudomonadales, Oceanospirillales, Enterobacterales, Xanthomonadales, Nevskiales and Chromatiales with averages of 13,457, 5914, 2558, 1970, 1453, and 654 reads, respectively. Phytopathogenic bacteria of the genera *Pseudomonas* and *Pantoea* were migrated and deposited in the stalactite cores, signifying that the upper ground cave area was cover by crops and/or native plants.

### 3.6. Actinobacteria

Within the Actinobacteria, most phylotypes were classified under the orders Micrococcales (44% of Actinobacteria) with an average of 12,910 reads, Thermoleophilia (32% of Actinobacteria) with an average of 22,151 reads, Propionibacteriales (26% of Actinobacteria) with an average of 7496 reads, Acidimicrobiales (11% of Actinobacteria) with an average of 7757 reads, and Pseudonocardiales (7% of Actinobacteria) with an average of 1917 reads. Under the order Micrococcales, the most dominant genera were *Pseudarthiobacter* and *Oryzihumus,* with averages of 12,910 and 1299 reads, respectively. Under the order Thermoleophilia, the most dominant genus was *Gaiella,* with an average of 17,605 reads. Under the order Propionibacteriales, the most dominant genera were *Microlunatus*, *Nocardioides*, *Marmoricola*, and *Cutibacterium* with averages of 2665, 1276, 1269, and 551 reads, respectively. The dominant bacteria species were *Pseudarthiobacter* and *Gaiella.*

### 3.7. Firmicutes

Within the Firmicutes, most phylotypes were classified under the order Bacillales (88% of Firmicutes) with an average of 37,010 reads and the order Lactobacillales (12% of Firmicutes) with an average of 4917 reads.

### 3.8. Acidobacteria

Within the Acidobacteria, most phylotypes were unclassified, and only 8% with an average of 2297 reads were classified under the order Blastocatellales.

### 3.9. Bacteroidetes

Within the Bacteroidetes, most phylotypes were classified under the orders Flavobacteriales (24% of Bacteroidetes) with an average of 1810 reads, Cytophagales (23% of Bacteroidetes) with an average of 1750 reads, unclassified (15% of Bacteroidetes) with an average of 1152 reads, Saprospirales (3% of Bacteroidetes) with an average of 224 reads, and 934 reads (12% of Bacteroidetes) were classified in the family Sphingobacteriaceae.

### 3.10. Verrucomicrobia

Within the 6107 reads of Verrucomicroobia, most phylotypes were classified under the genera *Luteolibacterv* with an average of 359 reads, *Terrimicrobium* with an average of 286 reads, *Spartobacteria* with an average of 233 reads, and unclassified (85% of Verrucomicroobia) with an average of 5527 reads.

## 4. Discussion

The results above show that the most abundant phyla were Proteobacteria (24%), Actinobacteria (20%), Firmicutes (13%), Acidobacteria (8%), Bacterioidetes (2%), and Verrucomicrobia (2%). All phyla mentioned above have been reported as bacteria depending on the type of carbon source from the upper ground cave environment [44]. The predominance of Proteobacteria representatives in the stalactite core composition could be associated with their rapid growth rates in nutrient-rich environments (upper ground cave environment), constituting a proxy for the soil carbon input [45]. Bacteria appearances, such as *Sphingomonas*, at 1142 reads (*α*-Proteobacteria), are isolated from many different aquatic and terrestrial habitats; *Ralstonia,* at 120 reads (*β*-Proteobacteria), which cause bacterial wilt in a very wide range of host plants; *Nitrosomonas*, at 293 reads (β-Proteobacteria), playing an important role in providing nitrogen to plants [46]; and Actinobacteria and Firmicutes, being saprophytic organisms that take part in soil decomposition mainly from upper ground cave environment [47], are the most significant evidence that bacteria from the upper ground cave environment is stored inside the stalactite core. All the above indicate that there should be bacterial transport processes with the water movement from the upper ground cave environment through a dripping process, forming cave speleothems such as stalactites and stalagmites [6].

Our data show that the *α*-Proteobacteria phylotypes found in the stalactite belonged mainly to *Sphingomonads* (Sphingomonadales; Sphingomonadaceae), which are widely distributed in landscapes and have been isolated from many different aqueous and terrestrial habitats, as well as from plant root systems. As reported by many authors [48,49,50], these microorganisms carried in dripping water are involved in the formation of speleothems.

The presence of nitrogen-fixing phylotypes (Rhizobiales, at 8902 reads, 37% of *α*-Proteobacteria) found in the stalactite (Table 1) is the clearest and most distinct evidence that these phylotypes belong mainly to upper ground soil with specificity to plant roots and not to a cave’s internal environment. Thus, water flow, either from rain or from larger stagnant volumes from the upper ground environment, carried with it these bacteria towards the cave’s ceiling and to the entrapment inside the stalactite. As mentioned above, except for dripping water, there is significant evidence that bacteria of the order Rhizobiales, influenced by the neighboring rhizosphere community, affected not only upper ground rhizosphere soil but was also reported to have colonized many subterranean Etruscan tombs [51].

Moreover, a wide variety of Actinobacteria presented by the four orders Micrococcales, Propionibacteriales, Acidimicrobiales, Pseudonocardiales, and the class Thermoleophilia, suggest that they play an important role in the soil’s development (upper ground cave environment). The dominant bacteria species, *Pseudarthiobacter* (Micrococcales), at 12,910 reads and *Gaiella* (Thermoleophilia), at 17,605 reads, both reported as rhizosphere microbia or plant growth-promoting rhizobacteria [52,53,54,55,56], are also significant evidence of a bacterial transport process with the water movement from the upper ground cave environment, as mentioned above.

Apart from the *α*-Proteobacteria, other specific bacterial phylotypes found in the stalactite core, such as the phytopathogenic bacteria in the genera *Pseudomonas*, *Ralstonia*, and *Pantoea* at 3064 reads, 120 reads, and 739 reads, respectively, suggest that the upper ground cave area was covered by crops and/or native plants. Specifically, *Pantoea* was reported as an epiphyte or endophyte bacterium of plant roots of pineapple, mandarin and orange trees [41].

According to Yun et al. [6], bacterial species from the upper cave environment that were transferred with water movement to caves showed seasonal variations. Our analysis presents different bacteria phylotypes reflecting the climate and season variations from the upper ground environment in the formation of the stalactite throughout the years. In detail, from the 18 Proteobacteria families, as shown in Table 1, Thioprofundaceae and Comamonadaceae showed clear evidence of seasonal variations. *Thioprofundum*: Thioprofundaceae (80 reads), which is a thermophilic genus [43] and *Polaromonas*: Comamonadaceae (487 reads,) which is a psychrophilic marine bacterium found on glacier surfaces in Antarctica [32,33] (Table 1), trapped inside the stalactite support the paleoclimate seasonal variations [57]. The bacterial genus *Polaromonas* seems to be among the predominant bacterial taxa in frozen ice since alignment sequencing was discovered by metagenomic studies of glacial environment globally, making *Polaromonas* one of the model taxons for researching microbial allocation configuration in the terrestrial cryosphere [33,58].

As the Agios Athanasios cave is characterized by darkness, no Cyanobacteria phyla were observed in the forming of stalactite. As heterotrophic organisms, Cyanobacteria can endure in dark caves and are eaten by cavern palpigrades [59]. It is fascinating that cyanobacterial species, as suggested by Barton and Jurado [60], adjust to the cave habitat by interacting with minerals there and some of the processes transforming the mineral structure of the cave walls, floors, and ceilings—for instance, by making speleothems such as stalactites and stalagmites [61]. One of the most intriguing problems, although not known exactly, is the beginning of the worldwide (cosmopolitan) allocation of cavernicolous cyanobacteria. Many studies [62,63] have reported cyanobacteria existing in both terrestrial sediments and aquatic cave environments around the globe. The total absence of cyanobacteria in our stalactite core samples may reveal difficult environmental conditions throughout the years for cyanobacteria to be formed and entrapped and that their presence during the formation of stalactites is not necessary at any point in time in the specific cave in Central Greece. The importance of cyanobacteria in the formation of cave speleothems may have to be reconsidered and to be researched further in the future.

Verrucomicrobia phylum (6107 reads), which also interestingly appeared in our research, were first observed in aquatic environments [64] but are now known to survive in extreme habitats [65,66]. They were found at freezing temperatures in the deep sea, in Antarctica, in moraine lakes, and in glacial meltwaters (5800 and 6350 m above sea level) in the remote Mount Everest region [66]. Moreover, a member of the Verrucomicrobia was also isolated from a hot spring (75–95 °C). Verrucomicrobia habitation inside the stalactite core further supports the paleoclimate seasonal variations presented in our research.

In addition, learning of the microbial’s rare biosphere and its abilities is applicable to approaching microbial ecology at quite a few levels—from host–microbiome relations [67,68,69] to microbial responses to climate change [70,71], to mention a few recent examples.

## 5. Conclusions

By gathering information on past climates and their impacts on ecosystems, scientists can develop and improve climate models that are used to predict future climates. Irrespective of their low relative plethora, newly released research proposes that rare microbial taxa may be more important to ecosystem functioning than previously expected [72]. Proof of the reaction of past ecosystems to a changing climate provides data on their adaptability and leads resource managers and other decision makers on actions to alleviate the repercussions of future climate change [67]. This could also serve as a Future Global Climate predictions model as climate change is predicted to impact regions differently, causing changes to the ocean, life, ice, and all other parts of the Earth’s system. Furthermore, paleomicrobiology research can focus on commensal microorganisms, species from non-human hosts, information from host-genomics, and the use of bacteria as proxies for additional information about past human health, behavior, migration, and culture [22].

In the present research, this bacterial data can shed light on the understanding of the microbial information entrapped in the stalactite for paleo-reconstruction, paleoecology, and paleoclimatology. Overall, our current results suggest that stalactites could serve as a bank source representing and storing microbe communities throughout the ages, from the upper or around cave surroundings, including soil, aquatic environment, plants, and animals.

## Figures and Tables

**Table 1:** The 18 families of Proteobacteria reported from the stalactite core sample and their environmental origin.

Phylum	Class	Order	Family (F)	Genus	Ecology	References
Proteobacteria (24% of Bacteria)	α-Proteobacteria (28% of Proteobacteria), 23,934 reads	Sphingomonadales (33% of α-Proteobacteria)	Sphingomonadaceae (85% of Sphingomonadales)	Sphingomonas (70% of F)	The sphingomonads are widely distributed in nature, having been isolated from many different aqueous and terrestrial habitats, as well as from plant root systems, clinical specimens, and other sources.	[28]
		Rhizobiales (37% α-Proteobacteria)	Bradyrhizobiaceae (31% of Rhizobiales)	unclassified	Bradyrhizobiaceae is a family presenting a broad taxonomic affiliation with organisms from different environments, such as soil, plant, or animal hosts.	
			Hyphomicrobiaceae	Pedomicrobium (11% of F)	Pedomicrobium is a ubiquitous bacterium dominant in biofilms of man-made aquatic environments such as water distribution systems.	[29]
			Rhizobiaceae (40% of Rhizobiales)	unclassified	Rhizobiaceae is a family of the Rhizobiales order in the Alphaproteobacteria class that presents genera associated with soil and planta hosts.	[30]
	β-Proteobacteria (21% of Proteobacteria) 17,931 reads	Burkholderiales (60% of β-Proteobacteria)	Oxalobacteraceae (31% of Burkholderiales)	Massilia (6% of F)	Massilia timonae gen. nov., sp. nov., isolated from blood of an immunocompromised patient with cerebellar lesions.	[31]
			Comamonadaceae (47% of Burkholderiales)	Polaromonas (9% of F)	Polaromonas vacuolata gen. nov., sp. nov., a psychrophilic, marine, gas vacuolate bacterium from Antarctica. Polaromonas is one of the most abundant genera found on glacier surfaces, yet its ecology remains poorly described.	[32,33]
			Burkholderiaceae (13% of Burkholderiales)	Ralstonia (8% of F)	Most common pathogens for causing nosocomial infections. It colonises the xylem, causing bacterial wilt in a very wide range of potential host plants.	[34]
	γ-Proteobacteria (40% of Proteobacteria), 34,298 reads	Pseudomonadales (39% of γ-Proteobacteria)	Moraxellaceae (79% of Pseudomonadales)	Acinetobacter (100% of F)	Acinetobacters are common, free-living saprophytes found in soil, water, sewage and foods.	[35]
			Pseudomonadaceae (24% of Pseudomonadales)	Pseudomonas (94% of F)	The members of the genus demonstrate a great deal of metabolic diversity and consequently are able to colonize a wide range of niches.	[36]
		Oceanospirillales (17% of γ-Proteobacteria)	Halomonadaceae (100% of Oceanospirillales)	Halomonas (99% of F)	Halomonas elongata, a new genus and species of extremely salt-tolerant bacteria.	[37]
		Xanthomonadales (11% of γ-Proteobacteria)	Xanthomonadaceae (92% of Xanthomonadales)	Lysobacter	The Lysobacter species live in soil, decaying organic matter, and fresh water.	[38]
			Rhodanobacteraceae (2% of Xanthomonadales)	unclassified		
		Enterobacterales (7% of γ-Proteobacteria)	Enterobacteriaceae (36% of Enterobacterales)	Escherichia/Shigella (94% of F)	A Common inhabitant of the gastrointestinal tract of humans and animals.	[39]
			Yersiniaceae (34% of Enterobacterales)	Serratia (100% of F)	Found in water, soil, plants, and animals. Some members of this genus produce a characteristic red pigment, Prodigiosin.	[40]
			Erwiniaceae (29% of Enterobacterales)	Pantoea (100% of F)	Pantoea species have been isolated from feculent material, in soil, water, plant (as epiphytes or endophytes), seeds, fruits (e.g., pineapple, mandarin oranges), and the human and animal gastrointestinal tracts, in dairy products, in blood and in urine.	[41]
		Nevskiales (4% of γ-Proteobacteria)	Steroidobacteraceae (49% of Nevskiales)	unclassified		
			Nevskiaceae (49% of Nevskiales)	Panacagrimonas (62% of F)	Soil bacteria.	[42]
		Chromatiales (2% of γ-Proteobacteria)	Thioprofundaceae (12% of Chromatiales)	Thioprofundum (100% of F)	Thermophilic chemolithoautotrophs from the deep sea.	[43]

## Data Availability

Not applicable.

## Supplementary Materials

Supplementary data are available at online at https://www.mdpi.com/article/10.3390/microorganisms9122411/s1. Figure S1: OTU, Figure S2: Class (16S-S1, 16S-S2, 16S-S3, 16S-S4), Figure S3: Family (16S-S1, 16S-S2, 16S-S3, 16S-S4), Figure S4: Genus (16S-S1, 16S-S2, 16S-S3, 16S-S4), Figure S5: Sample (Abundance—Phylum), Figure S6: Sample (Abundance—Class), Figure S7: Alpha diversity, Figure S8: Bray-Curtis Heatmap, Figure S9: Venn diagram, Figure S10: Krona pie chart.
