# Supplementary material for: Metataxonomic Analysis of Bacteria Entrapped in a Stalactite’s Core and Their Possible Environmental Origins
Source: Microorganisms. 2021 Nov 23;9(12):2411. doi: 10.3390/microorganisms9122411 (PMC8705861; doi:10.3390/microorganisms9122411)
Supplement: Supplementary file 1 [file microorganisms-09-02411-s001.zip › microorganisms-1458442-Supplementary S1-S10.pdf]

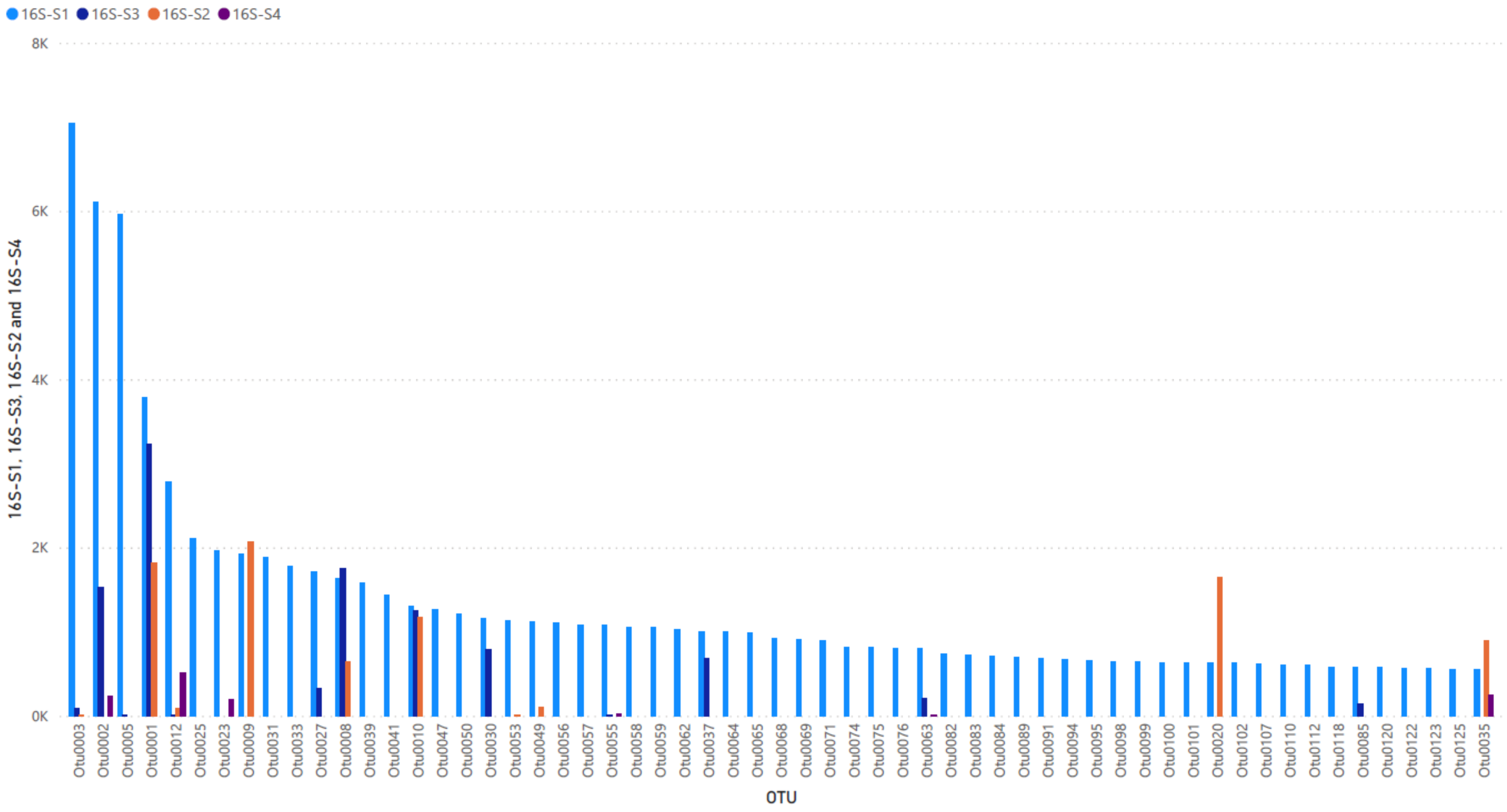

Figure S1. OTU

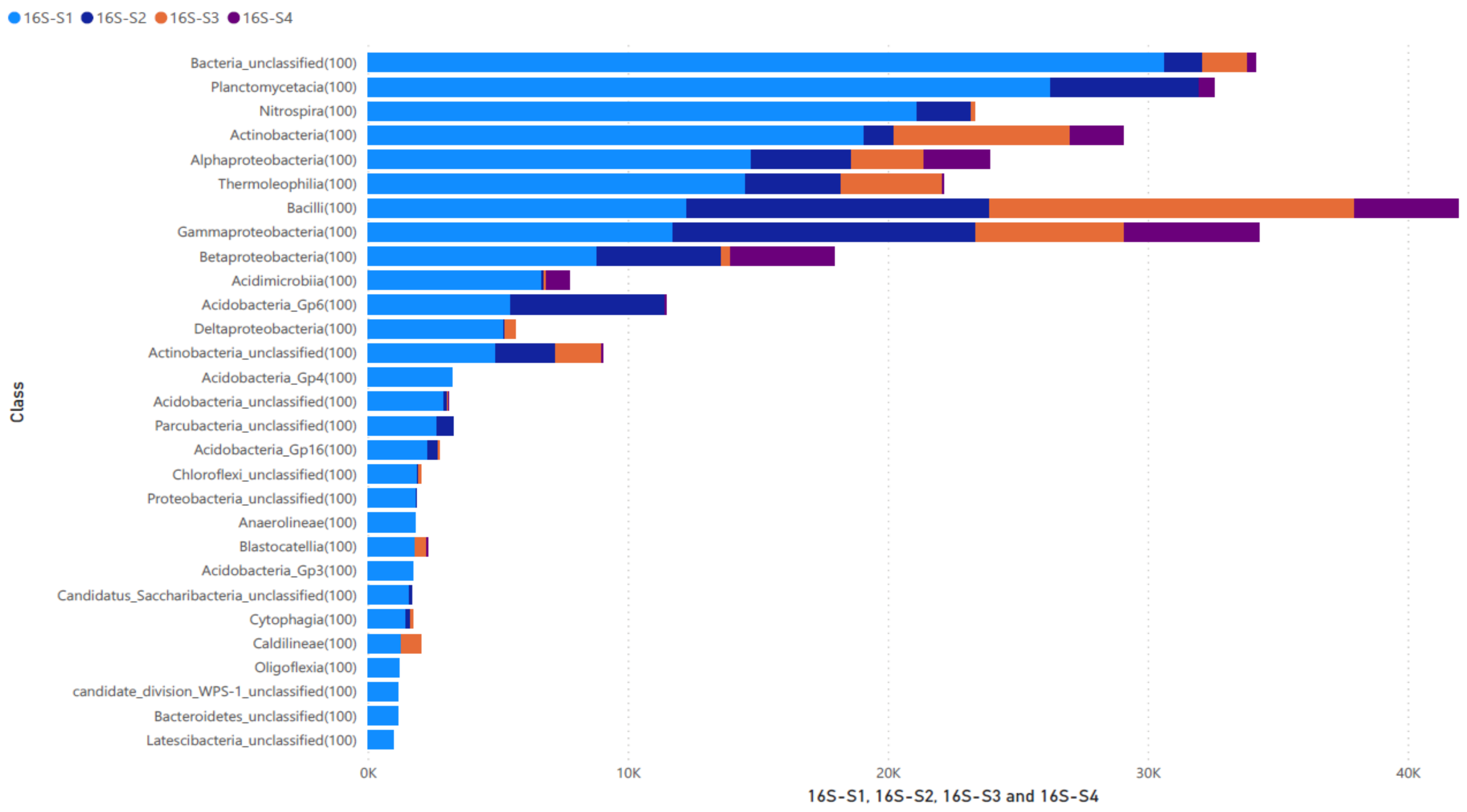

Figure S2. Class (16S-S1, 16S-S2, 16S-S3, 16S-S4)

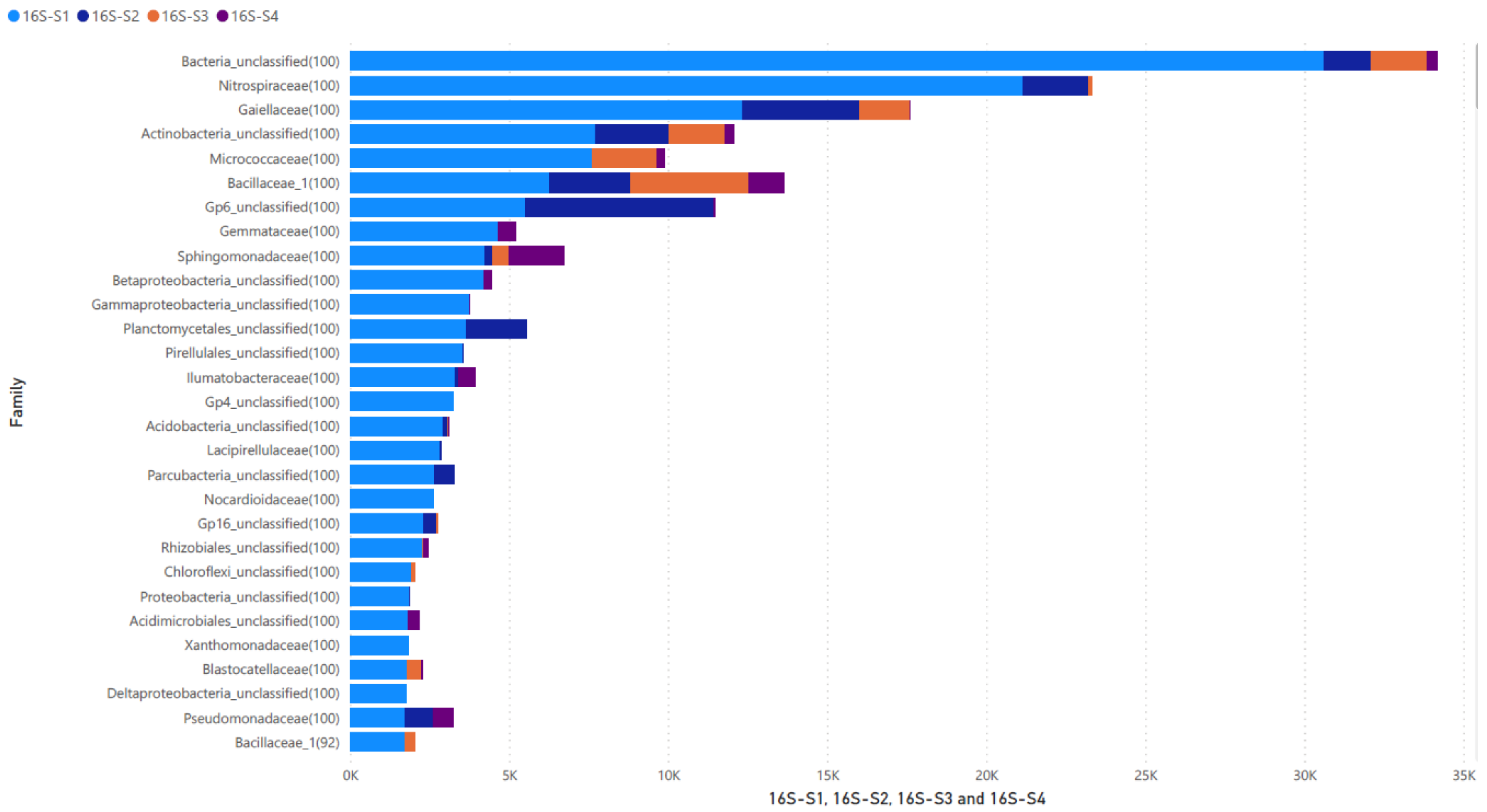

Figure S3. Family (16S-S1, 16S-S2, 16S-S3, 16S-S4)

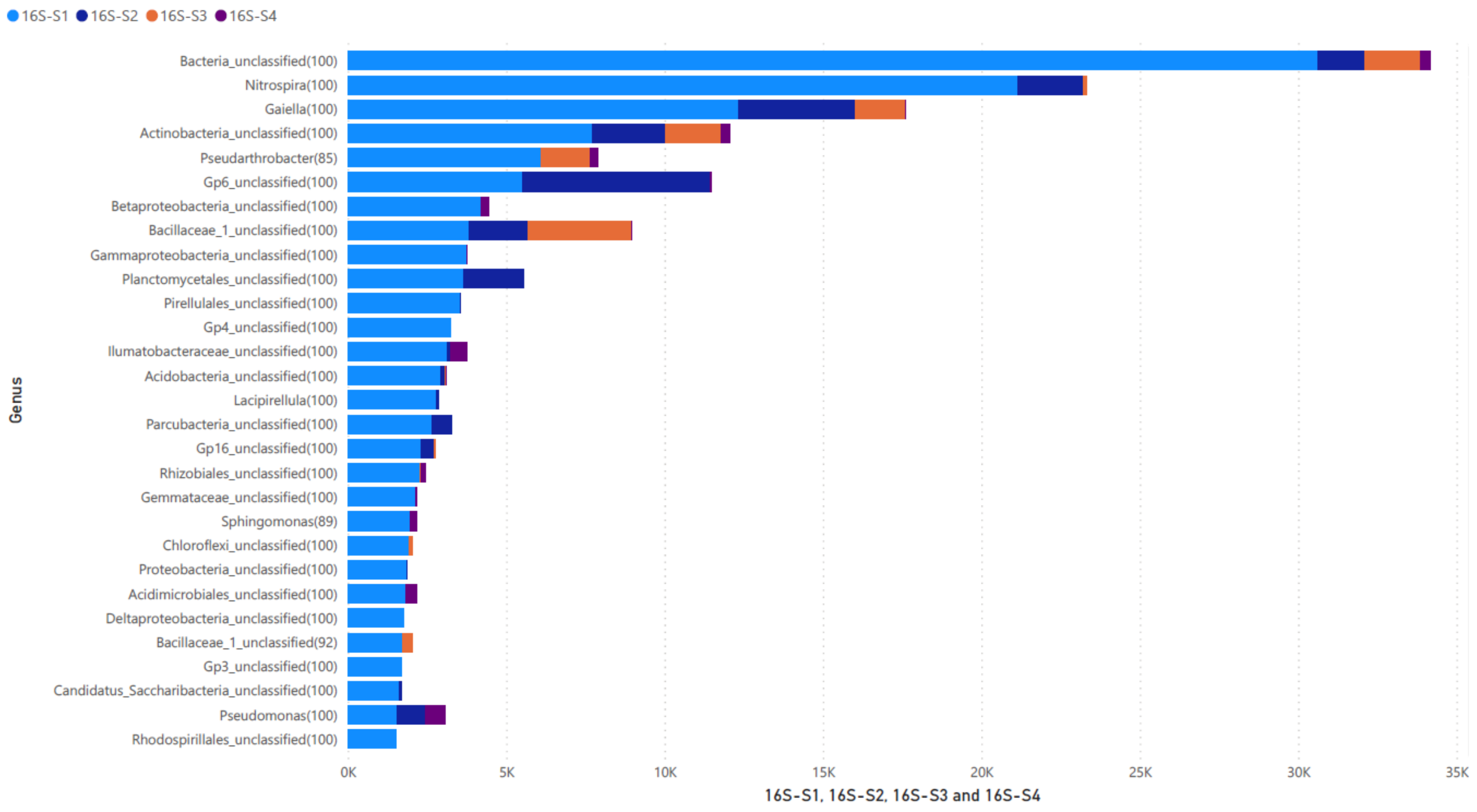

Figure S4. Genus (16S-S1, 16S-S2, 16S-S3, 16S-S4)

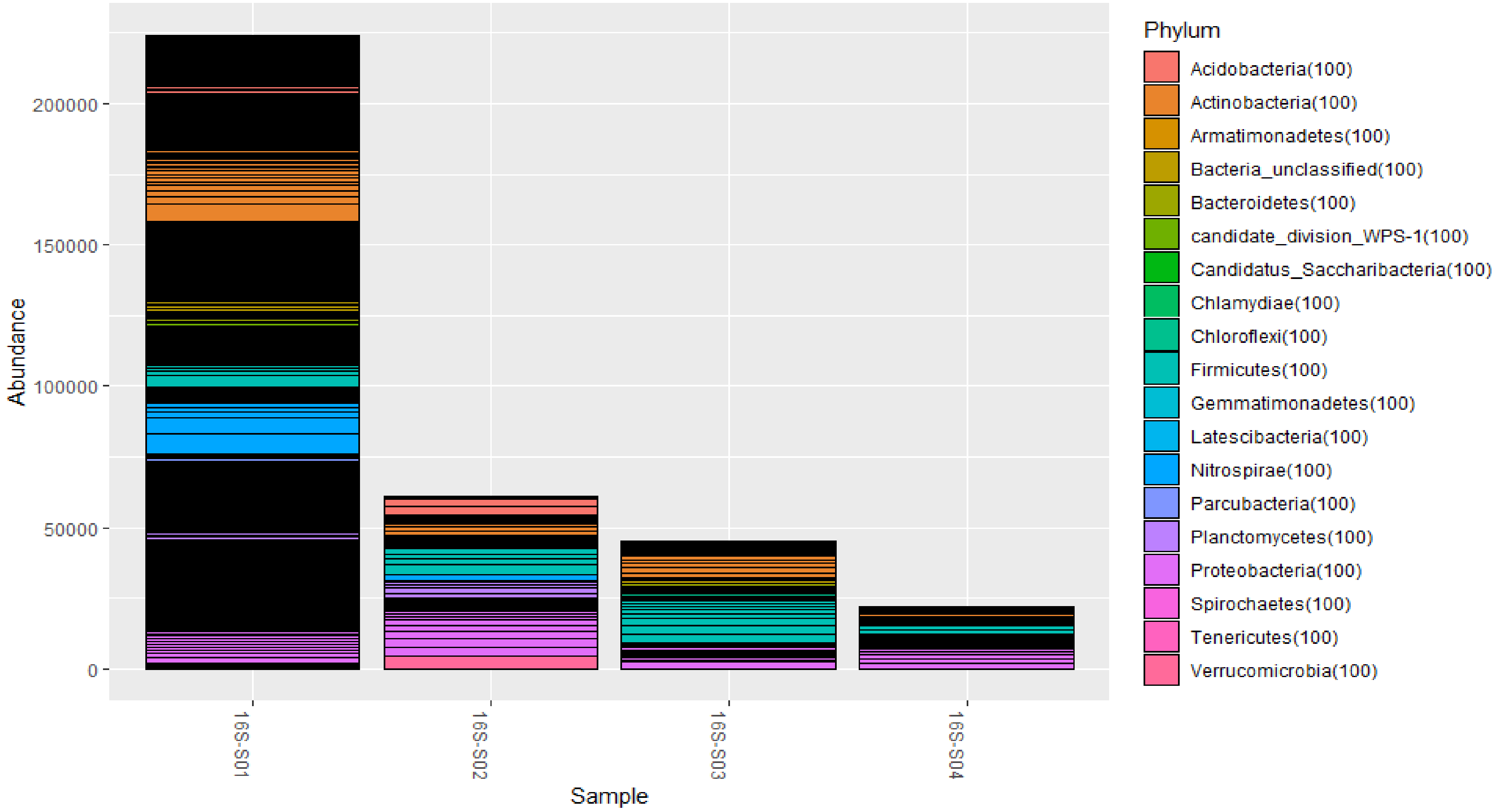

Figure S5. Sample (Abundance - Phylum)

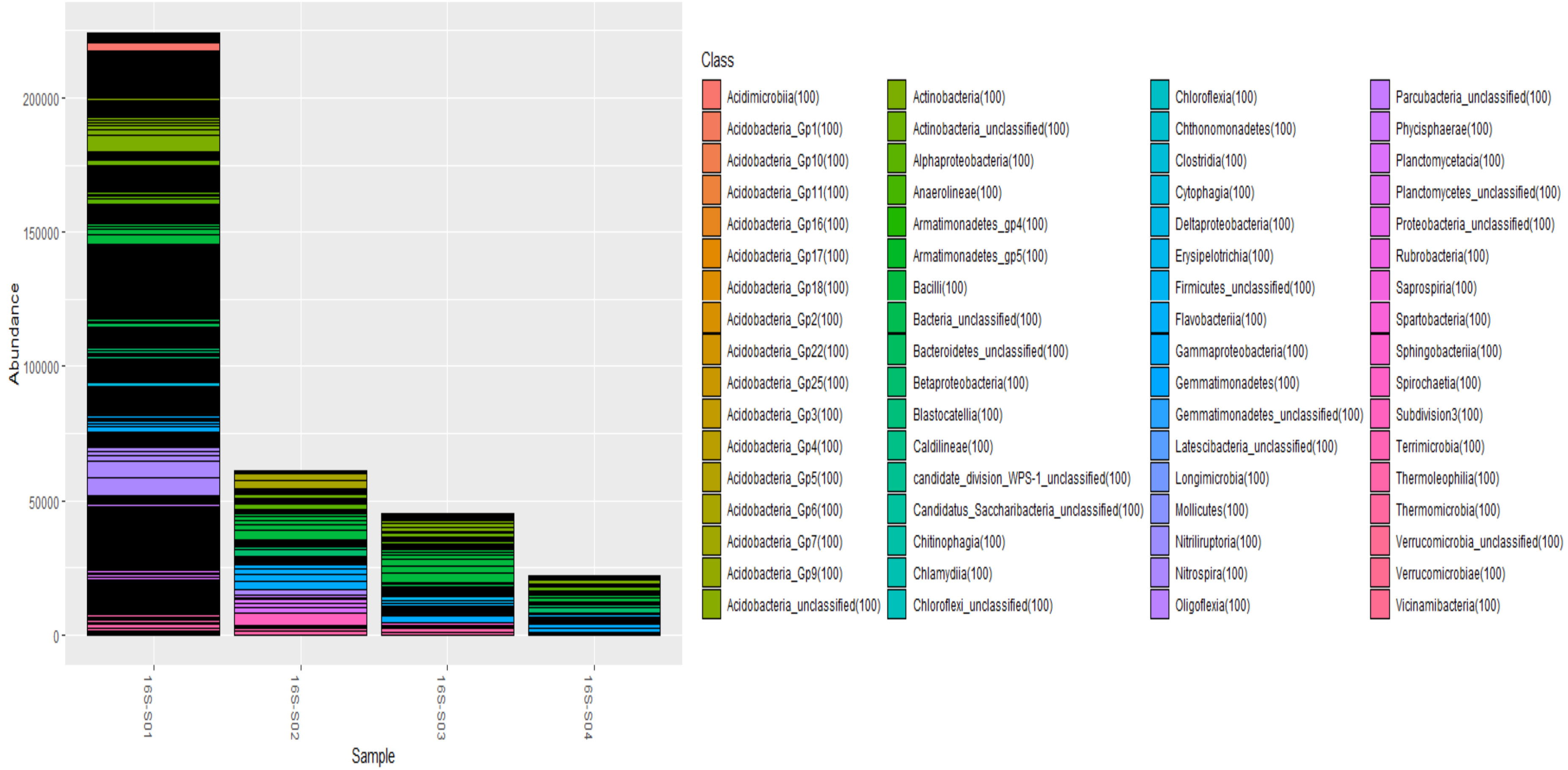

Figure S6. Sample (Abundance - Class)

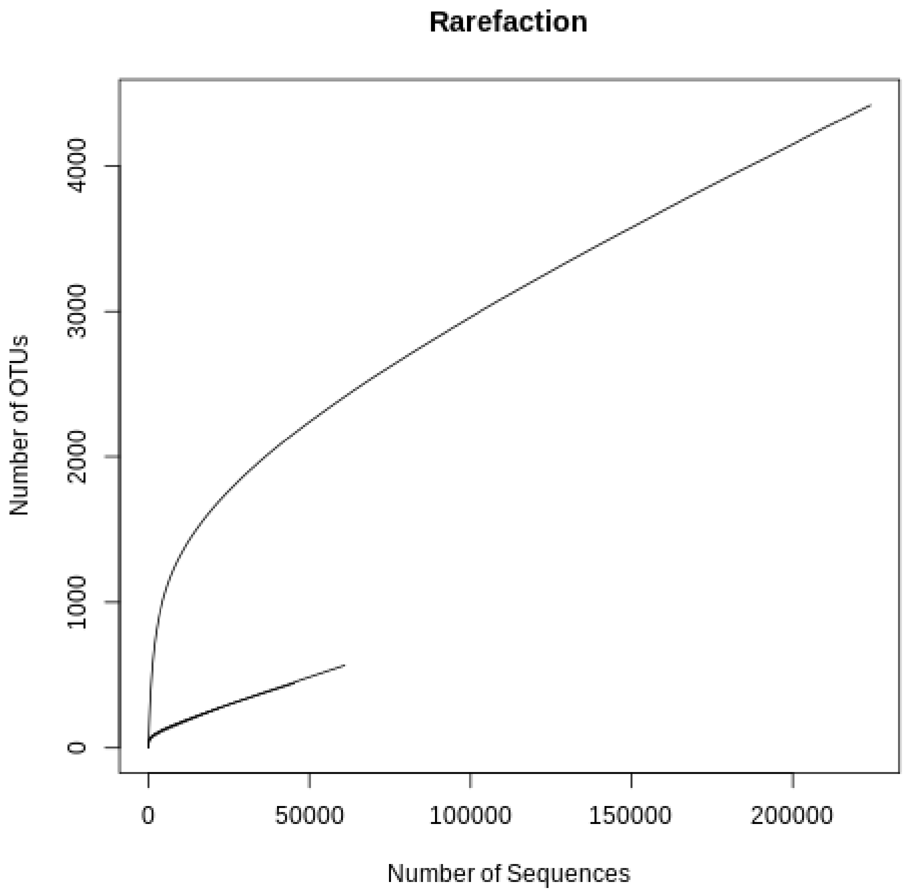

Figure S7. Alpha diversity

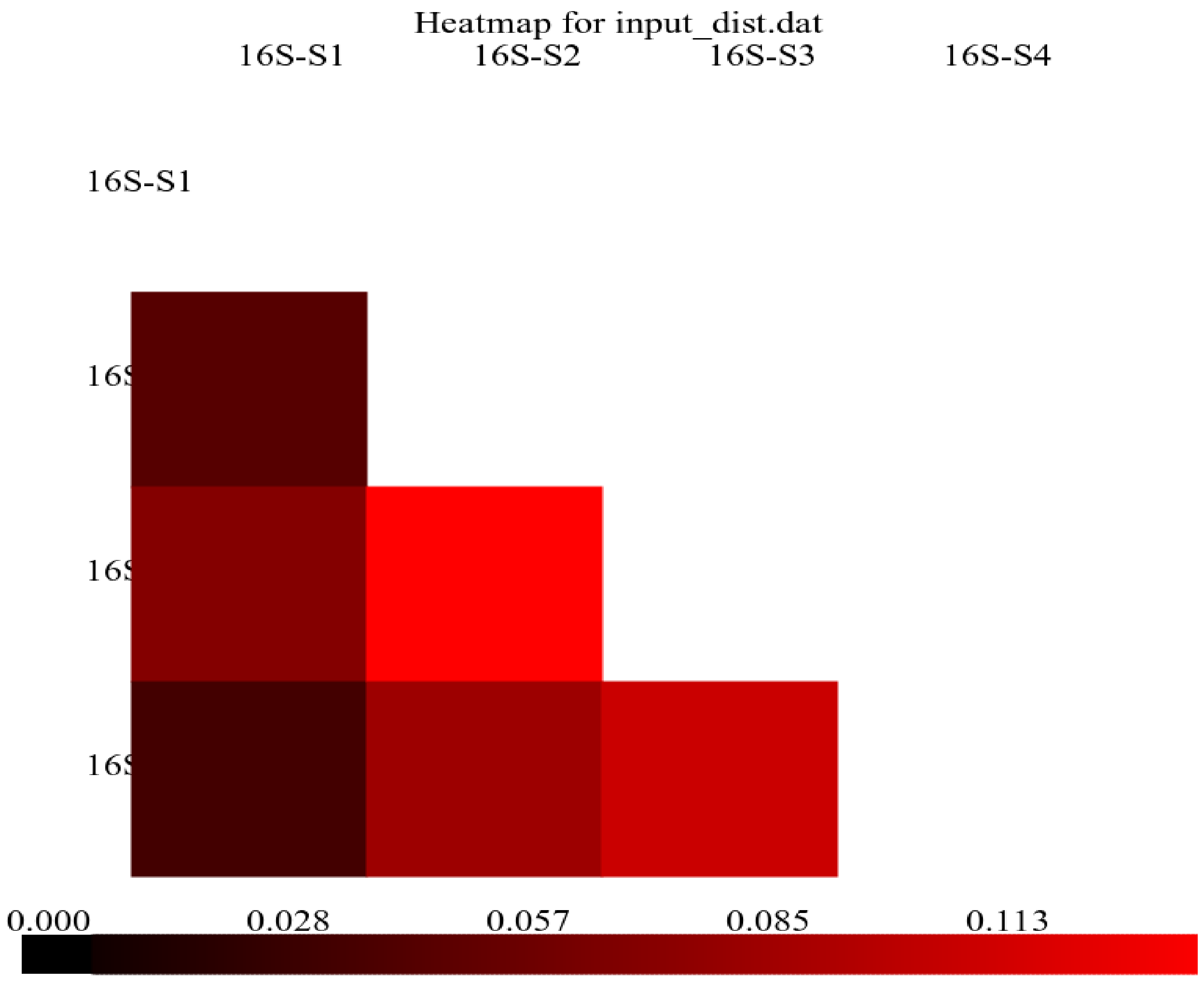

Figure S8. Bray-Curtis Heatmap

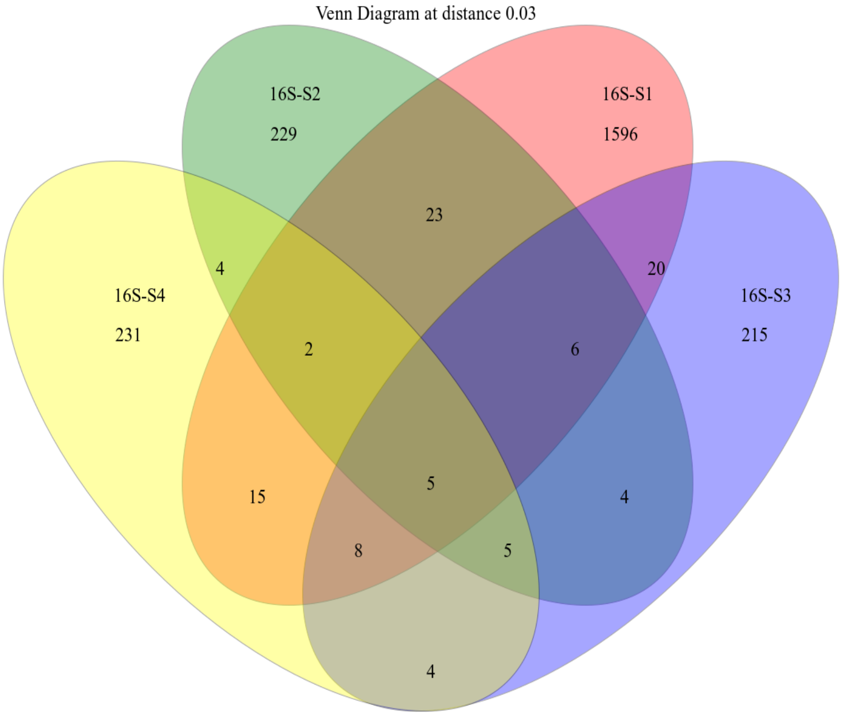

The number of species in group 16S-S1 is 1675
The number of species in group 16S-S2 is 278
The number of species in group 16S-S3 is 267
The number of species in group 16S-S4 is 274
The number of species shared between groups 16S-S1 and 16S-S2 is 36
The number of species shared between groups 16S-S1 and 16S-S3 is 39
The number of species shared between groups 16S-S1 and 16S-S4 is 30
The number of species shared between groups 16S-S2 and 16S-S3 is 20
The number of species shared between groups 16S-S2 and 16S-S4 is 16
The number of species shared between groups 16S-S3 and 16S-S4 is 22
The number of species shared between groups 16S-S1, 16S-S2 and 16S-S3 is 11
The number of species shared between groups 16S-S1, 16S-S2 and 16S-S4 is 7
The number of species shared between groups 16S-S1, 16S-S3 and 16S-S4 is 13
The number of species shared between groups 16S-S2, 16S-S3 and 16S-S4 is 10
The total richness of all the groups is 2367

Figure S9. Venn diagram

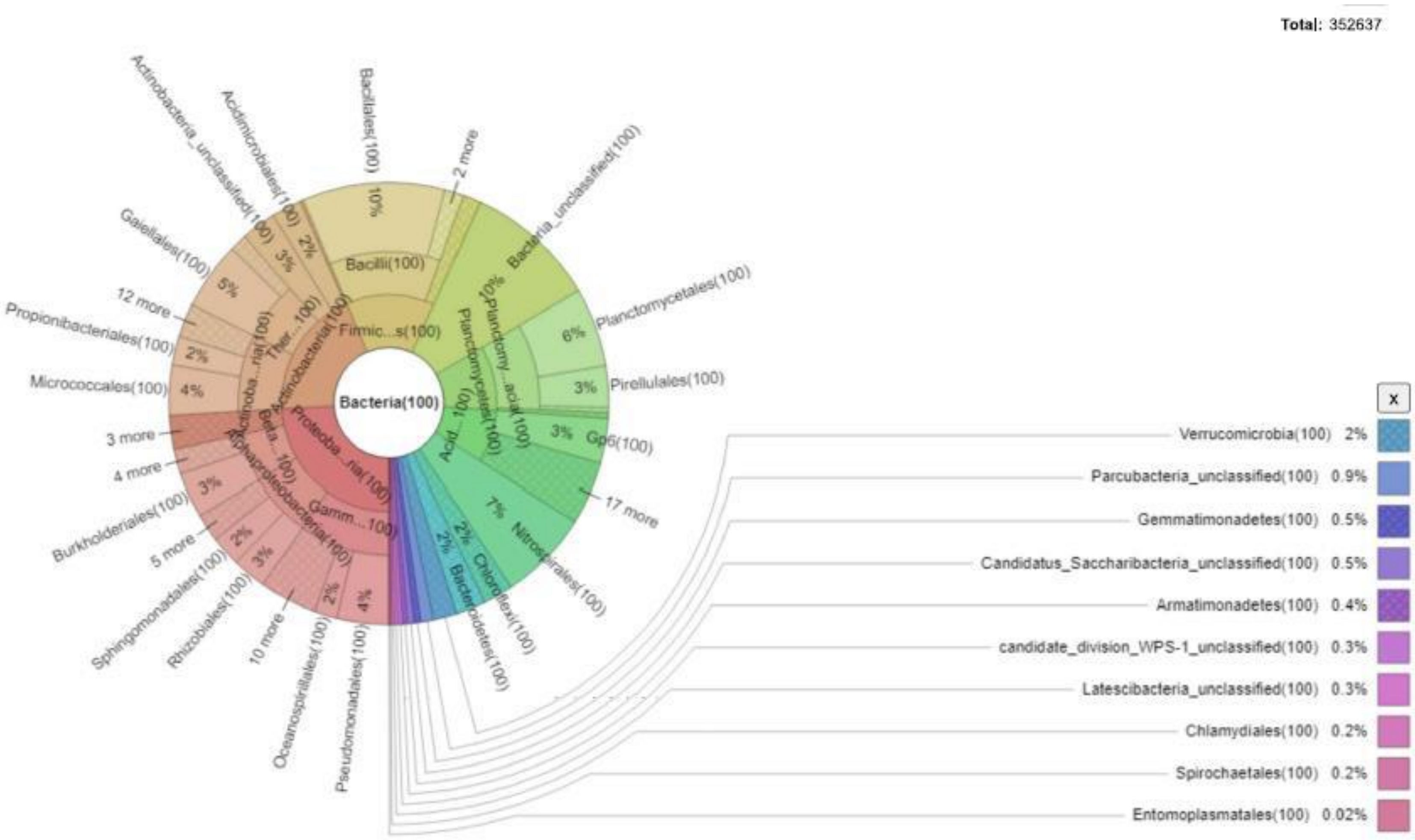

Figure S10. Krona pie chart
